# Rough colony morphology of *Mycobacterium massiliense* Type II genotype is due to the deletion of glycopeptidolipid locus within its genome

**DOI:** 10.1186/1471-2164-14-890

**Published:** 2013-12-17

**Authors:** Byoung-Jun Kim, Bo-Ram Kim, So-Young Lee, Yoon-Hoh Kook, Bum-Joon Kim

**Affiliations:** 1 Department of Microbiology and Immunology, Biomedical Sciences, Liver Research Institute, Cancer Research Institute and Seoul National University Medical Research Center (SNUMRC), Seoul National University College of Medicine, Seoul 110-799, Republic of Korea

**Keywords:** *Mycobacterium massiliense*, Glycopeptidolipid (GPL), Rough colony morphotype, GPL biosynthesis related genes, Comparative genomics

## Abstract

**Background:**

Recently, we introduced the complete genome sequence of *Mycobacterium massiliense* clinical isolates, Asan 50594 belonging to Type II genotype with rough colony morphology. Here, to address the issue of whether the rough colony morphotype of *M. massiliense* Type II genotype is genetically determined or not, we compared polymorphisms of the glycopeptidolipid (GPL) gene locus between *M. massiliense* Type II Asan 50594 and other rapidly growing mycobacteria (RGM) strains via analysis of genome databases*.*

**Results:**

We found deletions of 10 genes (24.8 kb), in the GPL biosynthesis related gene cluster of Asan 50594 genome, but no deletions in those of other smooth RGMs. To check the presence of deletions of GPL biosynthesis related genes in *Mycobacterium abscessus* − complex strains, PCRs targeting 12 different GPL genes (10 genes deleted in Asan 50594 genome as well as 2 conserved genes) were applied into 76 clinical strains of the *M. abscessus* complex strains [54 strains (Type I: 33, and Type II: 21) of *M. massiliense* and 22 strains (rough morphoype: 11 and smooth morphotype: 11) of *M. abscessus*]. No strains of the Type II genotype produced PCR amplicons in a total of 10 deleted GPL genes, suggesting loss of GPL biosynthesis genes in the genome of *M. massiliense* type II genotype strains.

**Conclusions:**

Our data suggested that the rough colony morphotype of the *M. massiliense* Type II genotype may be acquired via deletion events at the GPL gene locus for evolutionary adaptation between the host and pathogen.

## Background

Rapidly growing mycobacteria (RGM) infections in immunocompetent persons, as well as in persons with predisposing factors or who are immunosuppressed, are being reported more frequently [1,2]. Particularly, of the RGMs, *Mycobacterium abscessus* − complex is commonly associated with wound infections and abscess formation and is the most frequent RGM causing chronic lung disease [3,4]. Recent application of the combinatorial taxonomy including biochemical tests, anti-microbial susceptibility test, and multi-locus sequencing approach have suggested that the *M. abscessus* − complex is actually subdivided into three species: *Mycobacterium abscessus* subsp. *abscessus, M abscessus* subsp. *massiliense,* and *M. abscessus* subsp. *bolletii*; which exhibit clinically relevant differences in their antibiotic sensitivity profiles [5-7]. In South Korea, infection by members of the *M. abscessus* − complex is the most prevalent of RGM infections and second to the *M. avium* − complex of non-tuberculous mycobacteria (NTM) [8].

NTM has long been known to have both rough and smooth colony phenotypes [9,10]. This may be due mainly to the expression levels of glycopeptidolipids (GPLs). GPLs are produced by several NTMs, including RGMs, such as *M. abscessus*, *M. chelonae* and *M. smegmatis*, [11-13] and *M. avium* − complex (MAC) members, such as *M. avium* and *M. intracellulare*[14-16]. GPLs are responsible for smooth colonies and contribute to colonization of NTMs in the environment via biofilm formation; while, loss of GPLs is correlated with rough colonies and facilitates survival in macrophages [17].

In the *M. abscessus* − complex strains, smooth phenotypes have occasionally spontaneously reverted to rough morphotypes after several passages on agar plates or via in vivo passage into mice [17]. It was reported that there is a positive correlation between colony morphology and virulence, with rough variants generally being more virulent than smooth variants [17,18]. This may be due primarily to the reduced expression of GPL, resulting in excessive secretion of TNF-α by the macrophage [18]. Recently, targeted deletion of a gene, *mmpL4b*, in the *M. abscessus* is also reported to lead to loss of GPL and to show enhanced invasive abilities [19].

A recent molecular epidemiology study based on partial *hsp65* sequences (603 bp) indicated that *M. massiliense* (65/109 patients, 59.6%) of *M. abscessus* complex strains was more prevalent than *M. abscessus* (44/109 patients, 40.4%) in South Korea [20]. Interestingly, infections in 30 of 65 Korean patients (46.2%) with *M. massiliense*, were found to be caused by a distinct Type II genotype not encountered in other areas [20].

The most characteristic feature of this novel genotype is that all its strains showed the rough colony morphotype. This suggests that its rough colony phenotype may be due to an irreversible genetic factor rather than the reversible spontaneous reversion from smooth to rough morphotype previously introduced as a major mechanism for acquisition of the rough phenotype in *M. abscessus* − complex strains [17].

The aim of this study was to prove our hypothesis that the rough colony phenotype of the new *M. massiliense* Type II genotype may be genetically determined. For this purpose, we first compared the GPL biosynthesis related gene loci of *M. massiliense* Asan 50594, belonging to the Type II genotype for which we recently provided a complete genome, with those of other RGMs [21]. Second, to check whether GPL deletion is distinct for *M. massiliense* Type II genotype of *M. abscessus* − complex strains, PCR assays for the detection of GPL deletions were applied to the *M. abscessus* complex clinical strains, including a variety of groups.

## Results

### Differences between GPL expression patterns of *M. massiliense* Type I and Type II strains

**Figure 1:**
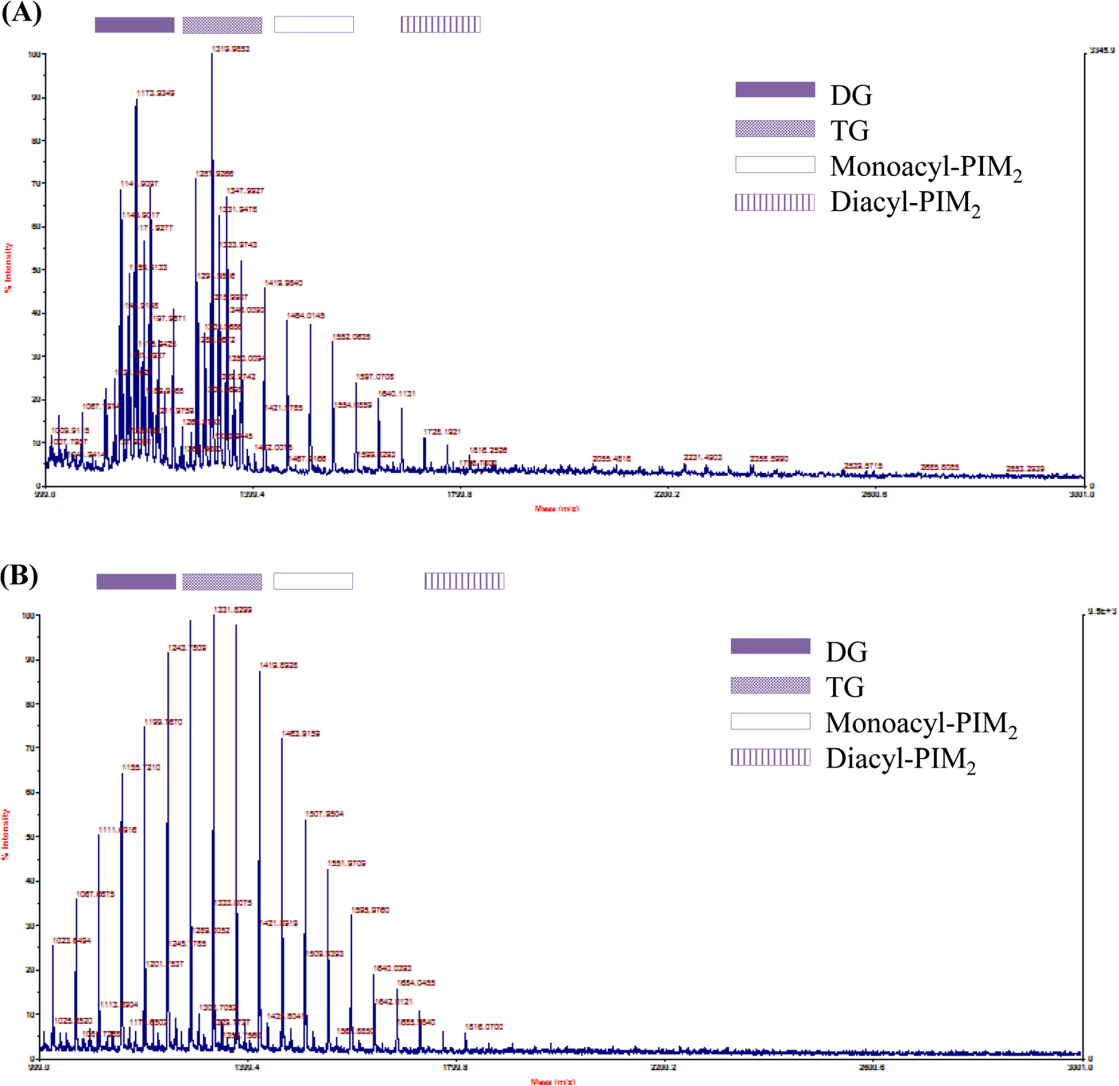
**MALDI-TOF MS analysis of extracted GPLs from: (A) *M. massiliense *Type I Asan 51843, (B) *M. massiliense *Type II Asan 50594.** Abbreviations: DG, diglycosylated GPLs; TG, triglycosylated GPLs; PIM2, phosphatidylinositol dimannoside.

To check out the differences in the GPL expression patterns of *M. massiliense* Type I and Type II strains, purified GPLs were examined and analyzed using matrix assisted laser desorption ionization-time of flight mass spectrometry (MALDI-TOF MS). Pronounced differences in the MALDI-TOF MS profiles were found between the GPLs of the two genotypes. In the case of *M. massiliense* Type I strain Asan 51843, the MALDI-TOF MS profiles showed two distinct clusters of peaks ranging from *m/z* 1101 to *m/z* 1245 and from *m/z* 1287 to *m/z* 1419, corresponding to diglycosylated GPL and triglycosylated GPL, respectively (Figure 1A). All four of the other Type I strains also showed MALDI-TOF mass spectrometry profiles similar to Strain 51843 (Additional file 1). However, the MALDI-TOF MS profiles of GPLs from *M. massiliense* Type II Asan 50594, were showed unusual, with significantly lower intensity of the peaks corresponding to the putative GPLs, compared to the profiles of the Type I strains (Figure 1B). Also, all four of the other Type II strains showed MALDI-TOF MS profiles similar to Strain 50594 (Additional file 1). This means that there was loss of GPLs in the cell wall components of the *M. massiliense* Type II strains.

### Comparative genomic analysis of the GPL biosynthesis related locus

To check out whether the GPL loss in *M. massiliense* Type II is genetically determined or not, we performed comparative genomic analysis of 29 GPL biosynthesis related genes (one larger cluster of ~40 kbp covering 19 genes, one smaller cluster of ~19 kbp covering 6 genes and four distributed genes in *M. abscessus*) from *M. massiliense* Type II Asan 50594 (GenBank Accession No., CP004374) and four other RGMs, [*M. abscessus* CIP 104536^T^ (GenBank Accession No., NC_010397), *M. massiliense* CCUG 48898^T^ (GenBank Accession No., AKVF00000000), *M. chelonae* CIP 104535^T^ (GenBank Accession No., AM231610-AM231615), and *M. smegmatis* str MC^2^ 155 (GenBank Accession No., AY439015)] [13,21,22].

**Figure 2:**
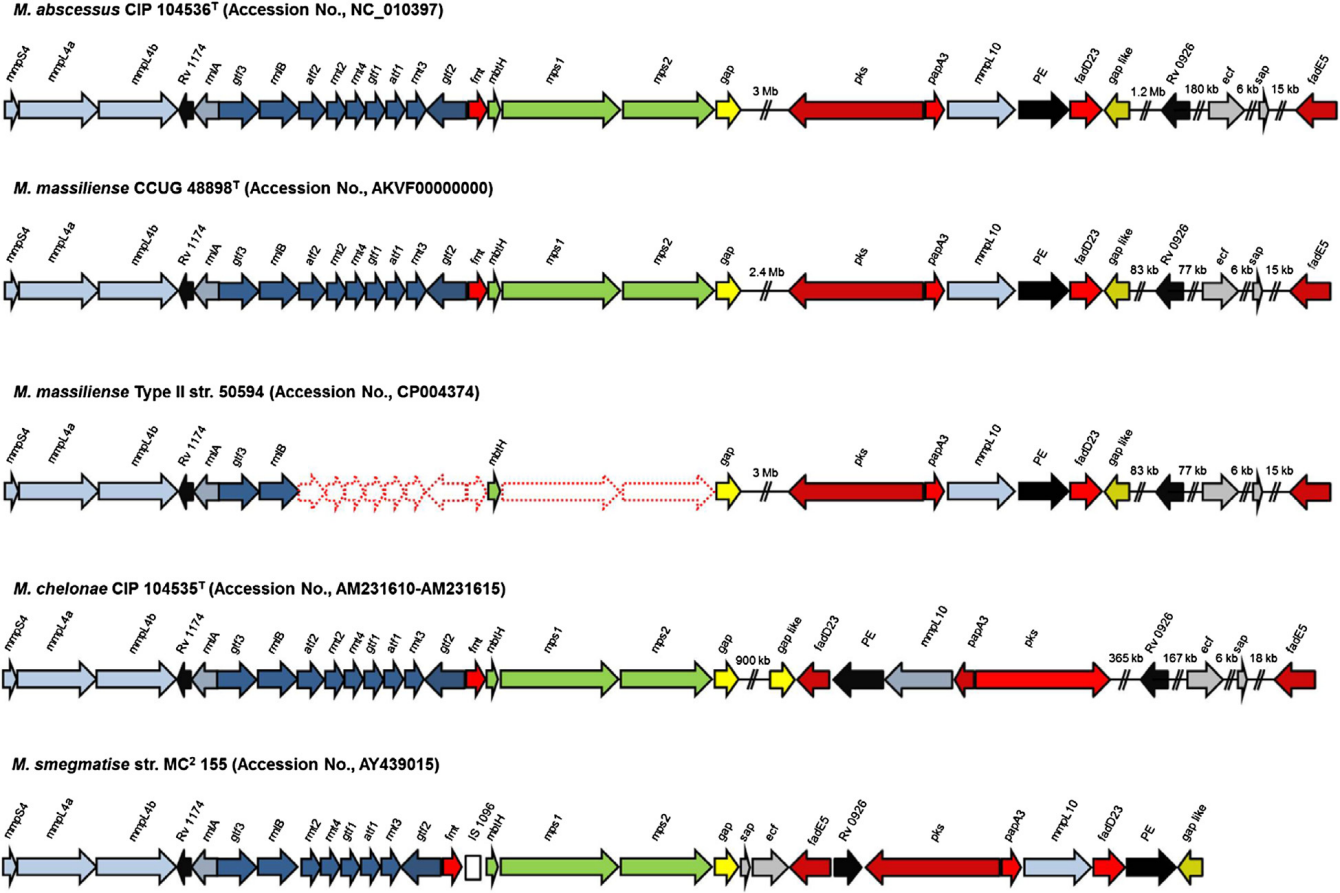
**Organization of GPL biosynthesis related genes.** Color code: Light blue – mmpL family; black – unknown; purple – sugar biosynthesis, activation, transfer and modifications; red – lipid biosynthesis, activation, transfer and modifications; green – pseudopeptide biosynthesis; yellow – required for GPL transport to the surface; grey – regulation.

**Figure 3:**
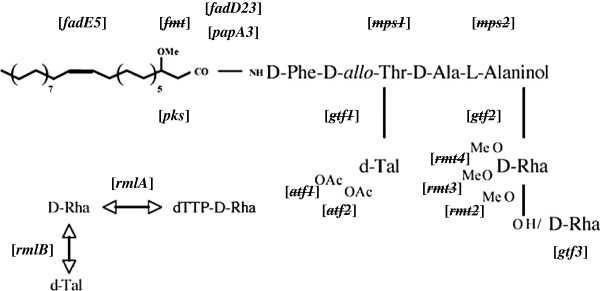
**Schematic representation of the structure of the GPLs from *M. smegmatis *(13).** The genes involved in GPL synthesis are indicated in brackets and the genes deleted in *M. massiliense* Type II Asan 50594, are indicated with strikethrough line. Abbreviations: OAc, acetyl; OMe, methyl; dTal, 6-deoxytalose; D-Rha, rhamnose of the D series.

When compared with *M. abscessus* CIP 104536^T^ and *M. massiliense* CCUG 48898^T^, or *M. massiliense* Type II Asan 50594, the percentage of identity of amino acids between 2 strains ranges between 86 and 100% (Table 1). Similar to the *M. abscessus* CIP 104536^T^, *M. massiliense* CCUG 48898^T^ or *M. massiliense* Type II Asan 50594 have GPL biosynthesis related genes which were divided into two clusters. In the genome of *M. massiliense* Type II Asan 50594, one cluster of ~11 kbp covering 8 genes (from MASS_4108 to MASS_4116, counterparts for *gap* to *mmpS4* in *M. abscessus*), one cluster of ~19 kbp covering 6 genes (from MASS_0918 to MASS_0923, counterparts for *gap*-like to *pks* in *M. abscessus*) and five distributed genes (MASS_4474, 4488, 4493, 4660 and 4722, counterparts for *fadE5*, *sap*, *ecf*, *Rv0926* and *mbtH* in *M. abscessus*, respectively) were found among the GPL-biosynthesis related genes. Interestingly, compared to other RGMs, there are no counterparts of 10 GPL biosynthesis related genes in the genome of *M. massiliense* Type II Asan 50594 (Table 1, Figure 2). All the deleted genes were found in the region corresponding to the first larger GPL cluster in *M. abscessus* CIP 104536^T^ or *M. massiliense* CCUG 48898^T^. Genes *atf1* and *atf2*, which are responsible for acetylation [23]; *gtf1* and *gtf2*, which are involved in the glycosylation of the lipopeptide core [24,25]; *rmt2*, *rmt3*, and *rmt4*, which are involved in the *O*-methylation of the various hydroxyl groups of the rhamnosyl unit; *fmt*, which is also involved in the *O*-methylation of the lipid moiety [26-28]; and *mps1* and *mps2*, which are responsible for assembling the tripeptide-aminoalcohol moiety [29], were deleted from the GPL locus of *M. massiliense* Type II Asan 50594 (Table 1, Figure 2 and Figure 3).

**Table 1:** **GPL biosynthesis related genes of *M. abscessus***, ***M. massiliense***, ***M. massiliense*** type II, ***M. chelonae*** and ***M. smegmatis***

***M. abscessus***^**a**^			***M. massiliense***^**b**^		***M. massiliense *** **Type II**^**c**^		***M. chelonae***^**d**^		***M. smegmatis***^**e**^	
**Gene**	**Locus_tag**	**Description**	**Locus_tag**	**ID (%)**	**Locus_tag**	**ID (%)**	**Locus_tag**	**ID (%)**	**Locus_tag**	**ID (%)**
mmpS4	MAB_4117c	Putative membrane protein, MmpS family	MMAS_40600	100	MASS_4116	100	MC1618	95	MSMEG0373	78
mmpL4a	MAB_4116c	Putative membrane protein, MmpL	MMAS_40590	100	MASS_4115	100	MC1619	95	MSMEG0374	78
mmpL4b	MAB_4115c	Putative membrane protein, MmpL	MMAS_40580	99	MASS_4114	99	MC1620	92	MSMEG0375	76
Rv1174	MAB_4114c	Conserved hypothetical protein	MMAS_40570	99	MASS_4113	99	MC1621c	89	MSMEG0376	48
rmlA	MAB_4113	Glucose-1-phosphate thymidylyltransferase	MMAS_40560	100	MASS_4112	100	MC1622c	97	MSMEG0377	85
gtf3	MAB_4112c	Putative glycosyltransferase GtfA	MMAS_40540	98	MASS_4110	99	MC1623	88	MSMEG0378	70
rmlB	MAB_4111c	Putative epimerase/dehydratase	MMAS_40530	99	MASS_4109	99	MC1624	93	MSMEG0379	78
atf2	MAB_4110c	Probable acetyltransferase AtfA	MMAS_40520	97			MC1625	89		
rmt2	MAB_4109c	Putative methyltransferase	MMAS_40510	98			MC1626	82	MSMEG0380	72
rmt4	MAB_4108c	Methyltransferase MtfB	MMAS_40500	99			MC1627	96	MSMEG0381	83
gtf1	MAB_4107c	Glycosyltransferase GtfA	MMAS_40490	99			MC1628	92	MSMEG0382	76
atf1	MAB_4106c	Acetyltransferase	MMAS_40480	98			MC1629	89	MSMEG0383	72
rmt3	MAB_4105c	Methyltransferase MtfD	MMAS_40470	100			MC1630	92	MSMEG0384	81
gtf2	MAB_4104c	Putative glycosyltransferase GtfB	MMAS_40460	97			MC1631c	81	MSMEG0385	67
fmt	MAB_4103c	Probable methyltransferase	MMAS_40450	99			MC1632	85	MSMEG0386	68
mbtH	MAB_4100c	MbtH-like protein	MMAS_40430	99	MASS_4722	86	MC1635	100	MSMEG0387	91
mps1	MAB_4099c	Probable non-ribosomal peptide synthetase	MMAS_40410	99			MC1636	86	MSMEG0390	70
mps2	MAB_4098c	Probable peptide synthetase NRP	MMAS_40400	99			MC1637	91	MSMEG0392	72
gap	MAB_4097c	Conserved hypothetical protein	MMAS_40390	99	MASS_4108	99	MC1638	85	MSMEG0393	58
sap	MAB_4454c	Conserved hypothetical protein (DGPF family)	MMAS_43620	99	MASS_4488	98	MC1299	94	MSMEG0394	31
ecf	MAB_4459c	RNA polymerase sigma-70 factor, ECF subfamily	MMAS_43670	98	MASS_4493	96	MC1294	85	MSMEG0395	47
fadE5	MAB_4437	Probable acyl-CoA dehydrogenase FadE	MMAS_43460	99	MASS_4474	99	MC1318c	96	MSMEG0396	78
Rv0926	MAB_4633	Conserved hypothetical protein	MMAS_45360	99	MASS_4660	99	MC1136c	91	MSMEG0397	36
pks	MAB_0939	Probable polyketide synthase	MMAS_07820	99	MASS_0923	98	MC0819	90	MSMEG0398	79
papA3	MAB_0938c	Probable conserved polyketide synthase associated protein PapA3	MMAS_07800	99	MASS_0922	99	MC0818c	92	MSMEG0399	78
mmpL10	MAB_0937c	Putative membrane protein, MmpL family	MMAS_07790	99	MASS_0921	99	MC0817c	95	MSMEG0400	75
PE	MAB_0936c	Conserved hypothetical protein	MMAS_07780	99	MASS_0920	99	MC0816c	89	MSMEG0402	64
fadD23	MAB_0935c	acyl-CoA synthetase	MMAS_07770	99	MASS_0919	99	MC0815c	88	MSMEG0401	73
gap-like	MAB_0934	Conserved hypothetical integral membrane protein	MMAS_07760	98	MASS_0918	98	MC0814	88	MSMEG0403	54

^*a*^Accession number: NC_010397 (13).

^*b*^Accession number: AKVF00000000 (24).

^*c*^Accession number: CP004374 (20).

^*d*^Accession number: AM231610-AM231615 (13).

^*e*^Accession number: AY439015 (13).

Considering that GPLs are related to formation of smooth colonies, these deletions represent the phenotypic characteristics of *M. massiliense* Type II, which only occurred in rough colonies [20].

### PCR confirmation of GPL biosynthesis related genes from *M. massiliense* and *M. abscessus* clinical isolates

**Figure 4:**
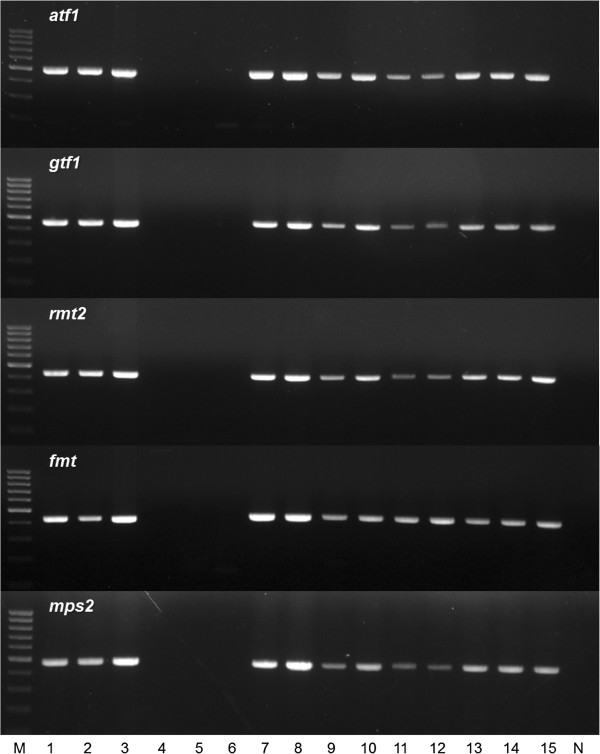
**Confirmation of deleted GPL biosynthesis related genes by PCR among clinically isolated *M. massiliense *and *M. abscessus.*** M, 100 bp DNA ladder; Lane 1, *M. massiliense* Type I Asan 51843; Lane 2, *M. massiliense* Type I Asan 50375; Lane 3, *M. massiliense* Type I Asan 15; Lane 4, *M. massiliense* Type II Asan 50594; Lane 5, *M. massiliense* Type II Asan 52012; Lane 6, *M. massiliense* Type II Asan 1; Lane 7, *M. massiliense* Type I (rough) Asan 22; Lane 8, *M. massiliense* Type I (rough) Asan 23; Lane 9, *M. massiliense* Type I (rough) Asan 54790; Lane 10, *M. abscessus* (smooth) Asan 57214; Lane 11, *M. abscessus* (smooth) Asan 57388; Lane 12, *M. abscessus* (smooth) Asan 58417; Lane 13, *M. abscessus* (rough) Asan 55088; Lane 14, *M. abscessus* (rough) Asan 56232; Lane 15, *M. abscessus* (rough) Asan 56544; N, negative control.

To check the presence of the GPL biosynthesis related genes from *M. massiliense* and *M. abscessus* clinical isolates, DNAs from 76 *M. abscessus* related strains were amplified by PCRs using 12 primer sets (Additional file 2), which targets 10 deleted genes in Asan 50594 genome: *atf1*, *atf2*, *fmt*, *gtf1*, *gtf2*, *rmt2*, *rmt3*, *rmt4*, *mps1*, and *mps2* and 2 conserved genes as PCR positive controls: *gap* and *rmlB* genes, which were found in the genome of all *M. abscessus* related strains including Asan 50594. Of 76 strains, 21 strains of *M. massiliense* Type II were not amplified by PCRs targeting 10 deleted genes in Asan 50594 genome, but amplified by PCRs targeting 2 conserved genes, suggesting the loss of corresponding GPL genes in all 21 *M. massiliense* Type II strains. But, all the remaining 55 strains were positively amplified by PCRs targeting 10 deleted genes as well as 2 conserved genes, suggesting the presence of targeted GPL biosynthesis genes in their genome. It should be noted that all the 23 strains with rough colony morphotype (12 *M. massiliense* Type I and 11 *M. abscessus* strains) except for *M. massiliense* Type II produced positive amplifications in PCRs targeting 10 deleted genes, suggesting there may be the disparity between *M. massiliense* Type II and other related strains in mechanism leading to rough colony phenotype (Figure 4, Table 2 and Additional file 3).

**Table 2:** PCR results of deleted or conserved genes at the GPL biosynthesis related locus from ***M. massiliense*** Type I, ***M. massiliense*** Type II, ***M. massiliense*** Type I (rough colony morphology), and ***M. abscessus*** (smooth and rough colony morphology), strains

		**PCR results [No. (%)]**				
	**Genes**	***M. massiliense *** **Type I**	***M. massiliense *** **Type II**	***M. massiliense *** **Type I (rough)**	***M. abscessus *** **(smooth)**	***M. abscessus *** **(rough)**
Deleted genes in *M. massiliense* Type II strain	*atf1*	21/21 (100)	0/21 (0)	12/12 (100)	11/11 (100)	11/11 (100)
	*atf2*	21/21 (100)	0/21 (0)	12/12 (100)	11/11 (100)	11/11 (100)
	*fmt*	21/21 (100)	0/21 (0)	12/12 (100)	11/11 (100)	11/11 (100)
	*gtf1*	21/21 (100)	0/21 (0)	12/12 (100)	11/11 (100)	11/11 (100)
	*gtf2*	21/21 (100)	0/21 (0)	12/12 (100)	11/11 (100)	11/11 (100)
	*mps1*	21/21 (100)	0/21 (0)	12/12 (100)	11/11 (100)	11/11 (100)
	*mps2*	21/21 (100)	0/21 (0)	12/12 (100)	11/11 (100)	11/11 (100)
	*rmt2*	21/21 (100)	0/21 (0)	12/12 (100)	11/11 (100)	11/11 (100)
	*rmt3*	21/21 (100)	0/21 (0)	12/12 (100)	11/11 (100)	11/11 (100)
	*rmt4*	21/21 (100)	0/21 (0)	12/12 (100)	11/11 (100)	11/11 (100)
Conserved genes	*gap*	21/21 (100)	21/21 (100)	12/12 (100)	11/11 (100)	11/11 (100)
	*rmlB*	21/21 (100)	21/21 (100)	12/12 (100)	11/11 (100)	11/11 (100)

## Discussion

Infection by *M. massiliense* strains of RGMs has gained importance together with its increasing prevalence over the world [30-35]. In particular, a recent study based on whole genome sequencing revealed the first evidence of human to human transmission in NTM infection, by proving its transmission between cystic fibrosis patients; suggesting unusually high infectivity of humans by *M. massiliense* strains [36]. In South Korea, a distinct epidemiologic trend was reported [37], of higher prevalence of *M. massiliense* strains (of the *M. abscessus* − complex); part of which may be attributed to the presence of the *M. massiliense* Type II genotype found only in Korean patients [20].

The combination of our genomic and molecular epidemiologic data in this study proved that all the strains belonging to a novel *M. massiliense* Type II genotype, showed loss of genes related to GPL biosynthesis (10 of 29 consecutive genes in *M. abscessus*) (Table 1, Figure 2 and Figure 3), resulting in irreversible rough phenotypes. Our PCR data targeting 12 GPL biosynthesis genes suggested that there may be the disparity between 2 groups, *M. massiliense* Type II and other *M. abscessus* related strains in evoking rough colony phenotypes. Unlike the former acquiring rough phenotype via a genetically determined mechanism, it cannot be excluded that GPL loss of the latter may be mediated by a not-yet determined non-genetic mechanism leading to the transient GPL loss, as reported in other papers [17]. But, the exact mechanism related to rough colony phenotype of the latter has to be elucidated in the future.

To our knowledge, this is the first report regarding this genetic defect of GPL biosynthesis in NTMs. Unlike for the *M. tuberculosis* − complex strains capable of transmission from human to human, in general, NTMs can infect humans only from environmental sources, although infection between cystic fibrosis patients by *M. abscessus* − complex strains has recently been reported [36]. Therefore, GPL is generally necessary for NTM survival in the natural environment (soils and water), and for human infection from environmental sources [17,23]. Particularly, in *M. abscessus* strains, the change of colony morphology from smooth to rough type, which provides an advantage for survival within a host macrophage, has so far been reported to happen spontaneously after host infection by reduction of GPL expression; not by irreversible genetic loss [17,36].

Given that *M. tuberculosis* − complex strains are strict pathogens that do not harbor gene loci related to GPL biosynthesis within their genomes, it is inferred that strains belonging to the *M. massiliense* Type II genotype may be more adapted to human infection than other members of *M. abscessus* complex. After sub-cultures of more than 10 generations on 7H9 broth or 7H10 agar plates, the reversion of rough to smooth type was not found in any Type II strains (data not shown). This further supports our hypothesis that the rough morphotype of Type II, like *M. tuberculosis*, may be an innate (genetic) trait derived from a smooth strain by evolutionary events, rather than a transient trait acquired during an *in vivo* infection.

Collectively considering our data only for the selective separation of the *M. massiliense* Type II genotype, we recommended combinatorial PCRs targeting both GPL deletion and conserved genes (e.g., *hsp65*), because they can be used for simple separation of the genotypes without additional procedures such as sequencing or PCR restriction analysis.

## Conclusions

In conclusion, our data showed that the *M. massiliense* Type II genotype showed gene loss related to GPL biosynthesis within its genome, resulting in a rough colony phenotype. To our knowledge, this is the first report of an NTM with a rough colony phenotype genetically determined by GPL gene loss.

## Methods

### Bacterial strains

All clinically isolated *M. abscessus* and *M. massiliense*[20] used in this study were cultured in 7H9 broth supplemented with 10% ADC at 37°C for 3 days. For MALDI-TOF analysis, five *M. massiliense* Type I (50375, 51843, 52352, Asan 7 and Asan 15) and five *M. massiliense* Type II (50594, 51048, 52012, Asan 1 and Asan 19) strains were used. For the PCR confirmation of GPL biosynthesis-related genes, a total of 76 strains [*M. massiliense* Type I (21 strains), *M. massiliense* Type II (21 strains), rough *M. massiliense* Type I (12 strains), smooth *M. abscessus* (11 strains) and rough *M. abscessus* (11 strains)] were used and listed in Table 3 and Additional file 4. All clinical strains were selected from among the 109 strains used in the previous report [20]. Separation of all *M. abscessus* − complex strains into genotypes or subspecies was performed by 603-bp *hsp65* based sequence analysis as described previously [20].

**Table 3:** **Clinically isolated *M. abscessus*** and ***M. massiliense*** used in this study

***M. massiliense***	
Type I	21
Type II	21
Type I (R)	12
***M. abscessus***	
Smooth	11
Rough	11
Total	76

Abbreviations: Type I, *M. massiliense* Type I strain; Type II, *M. massiliense* Type II strain; Type I (R), *M. massiliense* Type I (rough colony morphology) strain.

### Glycopeptidolipid (GPL) extraction

To characterize the GPL profiles of the *M. massiliense* Type I and Type II strains, five Type I (50375, 51843, 52352, Asan 7 and Asan 15) or Type II (50594, 51048, 52012, Asan 1 and Asan 19) strains were harvested at exponential phases of growth, and separated from the culture media by centrifugation at 4,000 rpm for 15 min. Each bacterial pellet was suspended in CHCl_3_/CH_3_OH [2:1, v/v; 10 ml/g (wet weight of bacterial pellet)]; sonicated two times (pulse for 1 min and stop for 10 sec, total 15 min), and incubated at 4°C overnight. After that, the suspensions were centrifuged to remove insoluble material and subjected to biphasic partitioning in CHCl_3_/CH_3_OH/H_2_O (4:2:1, v/v). Total lipid extracts were treated with an equal volume of NaOH (0.2 M in CH_3_OH, 45 min at 37°C), neutralized with glacial acetic acid, and dried in air. Finally, GPLs were extracted in CH_3_Cl/CH_3_OH (2:1, v/v) [17,38,39].

### Matrix-Assisted Laser Desorption Ionization-Time of Flight (MALDI-TOF) mass spectrometry analysis

MALDI-TOF mass spectrometry was performed on the extracted GPLs with a Voyager DE-STR MALDI-TOF instrument (Perseptive Biosystems) equipped with a pulse nitrogen laser emitting at 337 nm as previously described [24,40].

### Genome analysis of GPL biosynthesis related gene loci comparing *M. massiliense* Asan 50594 and other RGMs

The comparative genomic analysis was performed via comparison of pairwise alignments between the amino acid sequences of the GPL biosynthesis related 29 genes of *M. abscessus* CIP 104536^T^ (GenBank Accession No., NC_010397), *M. massiliense* CCUG 48898^T^ (GenBank Accession No., AKVF00000000), *M. massiliense* Type II Asan 50594 (GenBank Accession No., CP004374), *M. chelonae* CIP 104535^T^ (GenBank Accession No., AM231610-AM231615) and *M. smegmatis* str. MC^2^ 155 (GenBank Accession No., AY439015) [13,21,22]. The comparative genomic analysis was performed by pairwise alignments between the amino acid sequences of the GPL biosynthesis related genes of *M. massiliense* Type II Asan 50594 and the other RGMs mentioned above. Comparisons were performed using the MegAlign [41] and BLASTP program (http://blast.ncbi.nlm.nih.gov/Blast.cgi), and percentage of identities for genes were calculated.

### PCR applications targeting 12 GPL biosynthesis related genes

Total DNA was extracted from colonies using the bead beater-phenol extraction method, and then used as templates for PCR. To check the presence of GPL biosynthesis related genes in *M. abscessus* complex strains, we used purified DNAs from 76 clinical isolates, which included 33 *M. massiliense* Type I (21 strains: smooth, 12 strains:rough), 21 *M. massiliense* Type II (rough), and 22 *M. abscessus* (11 strains: smooth, 11 strains: rough) (Additional file 4). Using the *M. abscessus* CIP 104536^T^ (GenBank Accession No., NC_010397) and *M. massilense* CCUG 49989^T^ (GenBank Accession No., AKVF00000000) genome sequences [13,22], 12 primer sets were designed (Additional file 2). To amplify 12 independent genes from the DNA of *M. massiliense* and *M. abscessus* clinical isolates, the template DNA (50 ng) and 20 pmol of each primer were added into a PCR mixture tube (AccuPower PCR PreMix; Bioneer, Daejeon, South Korea) containing one unit of Taq DNA polymerase, 250 μM of deoxynucleotide triphosphate, 10 mM Tris–HCl (pH 8.3), 10 mM KCl, 1.5 mM MgCl_2_, and gel loading dye. The final volume was adjusted to 20 μl with distilled water, and the reaction mixture was then amplified as follows: denaturation at 95°C (5 min); 30 cycles of denaturation at 95°C (30 sec), annealing at 62°C (30 sec), elongation at 72°C (45 sec), and final elongation at 72°C (5 min) – using a model 9700 Thermocycler (Perkin-Elmer Cetus). Also, as a negative control, distilled water was amplified using all the primer sets. After amplification, the mixtures were electrophoresed in 1.5% agarose gel with GeneRuler™ 100 bp DNA ladder marker (Thermo Scientific, Pittsburgh, PA, United States).

### Availability of supporting data

The data sets supporting the results of this article are included within the article and its additional files (Additional files 1, 2, 3 and 4).

## Abbreviations

GPL: Glycopeptidolipid; RGM: Rapidly growing mycobacteria; NTM: Non-tuberculous mycobacteria; MAC: *Mycobacterium avium*-complex; MALDI-TOF: Matrix-assisted laser desorption ionization-time of flight; DG: Diglycosylated GPLs; TG: Triglycosylated GPLs; PIM2: Phosphatidylinositol dimannoside; OAc: Acetyl; OMe: Methyl; dTal: 6-deoxytalose; D-Rha: Rhamnose of the D series.

## Competing interests

The authors declare non-financial competing interests.

## Authors’ contributions

BJK (Byoung-Jun Kim) carried out MALDI-TOF and comparative genome analysis and interpretation the data. BRK and SYL carried out PCR and purification GPLs. YHK helped to draft the manuscript. BJK (Bum-Joon Kim) conceived of the study, participated in study design and drafted the manuscript. All authors read and approved the final manuscript.

## Supplementary Material

Additional file 1 **MALDI-TOF mass spectrometry profiles of GPLs from *M. massiliense *** **Type I and Type II strains: (A) *M. massiliense *** **Type I Asan 50375, (B) *M. massiliense *** **Type I Asan 52352, (C) *M. massiliense *** **Type I Asan 7, (D) *M. massiliense *** **Type I Asan 15, (E) *M. massiliense *** **Type II Asan 51048, (F) *M. massiliense *** **Type II Asan 52012, (G) *M. massiliense *** **Type II Asan 1, (H) *M. massiliense *** **Type II Asan 19.** Click here for file

Additional file 2 List of primers used in this study. Click here for file

Additional file 3 **Confirmation the deleted GPL biosynthesis related genes by PCR among clinical isolated *M. massiliense *** **and *M. abscessus.*** M, 100 bp DNA ladder; Lane 1, *M. massiliense* Type I Asan 51843; Lane 2, *M. massiliense* Type I Asan 50375; Lane 3, *M. massiliense* Type I Asan 15; Lane 4, *M. massiliense* Type II Asan 50594; Lane 5, *M. massiliense* Type II Asan 52012; Lane 6, *M. massiliense* Type II Asan 1; Lane 7, *M. massiliense* Type I (rough) Asan 22; Lane 8, *M. massiliense* Type I (rough) Asan 23; Lane 9, *M. massiliense* Type I (rough) Asan. 54790; Lane 10, *M. abscessus* (smooth) Asan 57214; Lane 11, *M. abscessus* (smooth) Asan 57388; Lane 12, *M. abscessus* (smooth) Asan 58417; Lane 13, *M. abscessus* (rough) Asan 55088; Lane 14, *M. abscessus* (rough) Asan 56232; Lane 15, *M. abscessus* (rough)Asan 56544; N, negative control. Click here for file

Additional file 4 List of clinical isolates used in this study. Click here for file
