# Supplementary figures and images for: Rough colony morphology of Mycobacterium massiliense Type II genotype is due to the deletion of glycopeptidolipid locus within its genome
Source: BMC Genomics. 2013 Dec 17;14:890. doi: 10.1186/1471-2164-14-890 (PMC3878547; doi:10.1186/1471-2164-14-890)

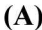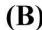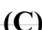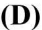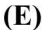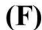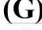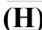

Supplement: Additional file 1 — MALDI-TOF mass spectrometry profiles of GPLs from M. massiliense Type I and Type II strains: (A) M. massiliense Type I Asan 50375, (B) M. massiliense Type I Asan 52352, (C) M. massiliense Type I Asan 7, (D) M. massiliense Type I Asan 15, (E) M. massiliense Type II Asan 51048, (F) M. massiliense Type II Asan 52012, (G) M. massiliense Type II Asan 1, (H) M. massiliense Type II Asan 19. [file 1471-2164-14-890-S1.pdf]

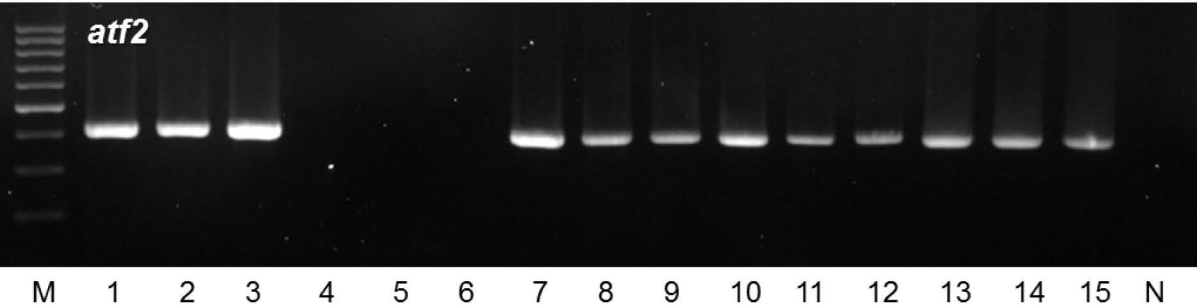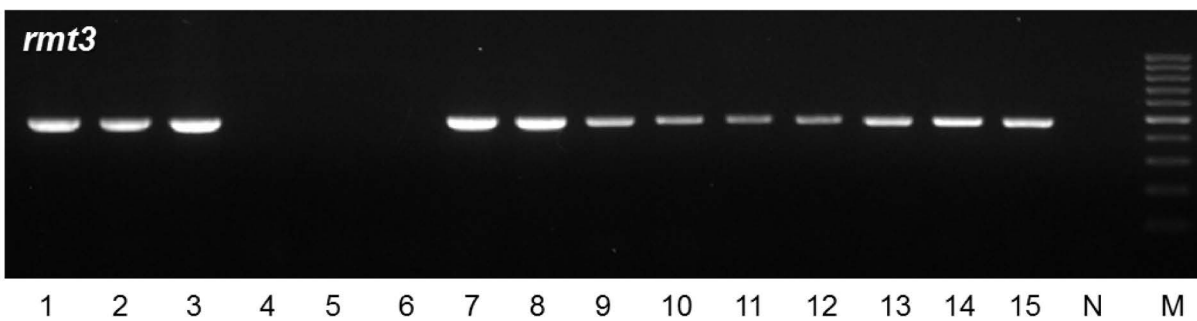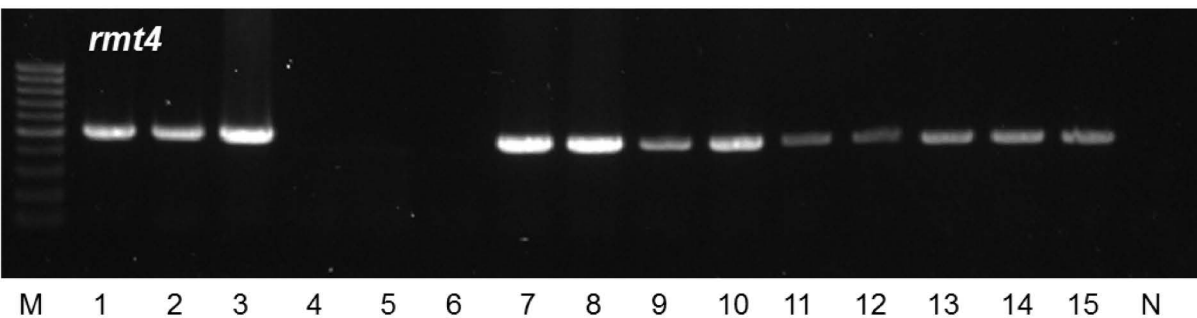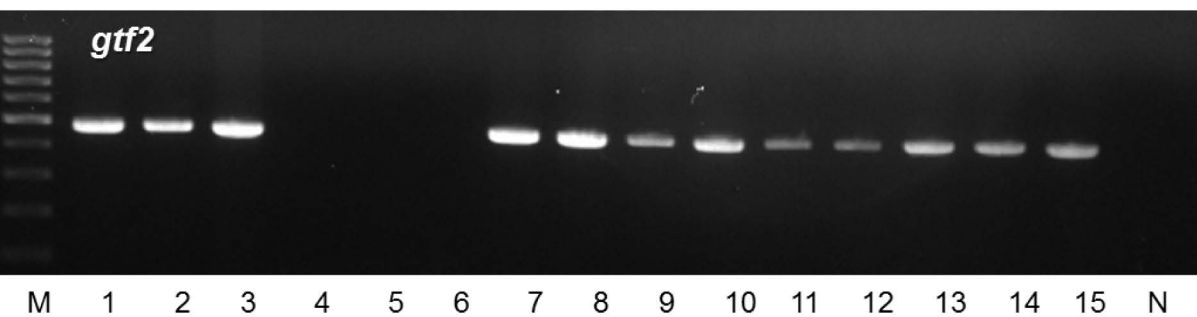

Supplement: Additional file 3 — Confirmation the deleted GPL biosynthesis related genes by PCR among clinical isolated M. massiliense and M. abscessus. M, 100 bp DNA ladder; Lane 1, M. massiliense Type I Asan 51843; Lane 2, M. massiliense Type I Asan 50375; Lane 3, M. massiliense Type I Asan 15; Lane 4, M. massiliense Type II Asan 50594; Lane 5, M. massiliense Type II Asan 52012; Lane 6, M. massiliense Type II Asan 1; Lane 7, M. massiliense Type I (rough) Asan 22; Lane 8, M. massiliense Type I (rough) Asan 23; Lane 9, M. massiliense Type I (rough) Asan. 54790; Lane 10, M. abscessus (smooth) Asan 57214; Lane 11, M. abscessus (smooth) Asan 57388; Lane 12, M. abscessus (smooth) Asan 58417; Lane 13, M. abscessus (rough) Asan 55088; Lane 14, M. abscessus (rough) Asan 56232; Lane 15, M. abscessus (rough)Asan 56544; N, negative control. [file 1471-2164-14-890-S3.pdf]
